# Denial of Personal Racial Discrimination and Its Impact Among People of Color Who Use Substances: Implications for Measuring Racial Discrimination in Substance Use Research

**DOI:** 10.1007/s40615-024-02033-w

**Published:** 2024-06-10

**Authors:** Hans Oh, Mojgan Sami, Brittany Blevins, Hannah Hanson, Emma Herzig, Catherine Ho, Ryan Lee, Kelly Wong, Jimi Huh

**Affiliations:** 1 University of Southern California, Los Angeles, USA; 2 California State University, Fullerton, USA

**Keywords:** Racial discrimination, Racism, Substance use, Focus groups

## Abstract

**Background:**

Even though racism is pervasive, some people of color may deny experiencing racial discrimination or may report being unaffected by it. This study examines the contexts and factors that may contribute to these responses among people who use substances.

**Methods:**

We conducted seven focus groups (5–9 participants per group, total *N* = 43) among Black, Latino, and Asian American adults between the ages of 21 to 44 years old who reported current use of two or more of the following substances: alcohol, cigarettes, e-cigarettes, or cannabis. Data were analyzed using reflexive thematic analysis.

**Results:**

Across all three ethno-racial groups, we found some respondents minimized or denied personal experiences of racial discrimination or hesitated to identify their experiences as racial discrimination, which in turn led to respondents to express uncertainty about seeing any sort of connection between racial discrimination and substance use. Themes included a minority comparison effect; a drowning out effect; diversity and racial composition of context; passing as White; and covertness of racism. Also, there were contradictions in accounts, and responses often depended on orienting cues.

**Conclusions:**

While researchers continue to find associations between racial discrimination and substance use, some people of color may not acknowledge this connection. Recommendations include aligning definitions of racism between academic and public/popular discourse; updating measures to keep up with the evolving forms of racism using context-specific examples; combining subjective measures of racial discrimination with objective measures of racism; and dialoguing with the public to raise awareness around how racism is defined.

## Introduction

In public and popular discourse, racial discrimination is often understood as the mistreatment perpetrated against a person or group of people because of their race. Often, the term “racial discrimination” evokes images of racial slurs or hate crimes that occur at an individual level. However, in academic and scholarly discourse, racial discrimination has long been understood as a part of a larger system of racism—a vast and interlocking system of power that privileges the dominant White group (that is, those with ancestral ties to Europe) while oppressing people of color [3, 5, 22, 49]. Williams and colleagues [49] identify domains of racism, including structural, cultural, and individual-level discrimination. This conceptualization acknowledges that racism is not merely a set of acontextual and apolitical individual experiences; rather, racism is an organized social system that inextricably links individual experiences to much larger social forces. These larger social forces intricately shape behavioral health outcomes and racial disparities through multiple entangled pathways [18, 19].

Gee and Hicken [16] argue that racial inequalities endure due to structural racism, which is a vast system of interconnected institutions operating within racialized rules that uphold White supremacy, allowing racism to shapeshift and re-emerge in new forms despite antiracist efforts directed at institutions. The authors underscore the importance of redirecting the focus away from specific individuals and institutions, and instead focusing on the connections across institutions. Along these lines, racism has been identified as a fundamental cause of health inequalities [37], shaping socioeconomic status, social position, opportunity structures, and resources, which in turn shape health outcomes. These conceptualizations of racism show a complicated system that occurs at all levels of society. As such, no measure can hope to capture the full scope, depth, and complexity of racism [30].

In recent decades, studies on substance use have focused on the role of self-reported experiences of racial discrimination. By and large, studies suggest that people who report more racial discrimination often have greater risk for substance use [17, 21, 41, 47]. Popular measures of discrimination used in these studies elicit the range and frequency of experiences of racial discrimination on a day-to-day basis [7]. However, studies have also examined more nuanced experiences of racial discrimination that are not as overt, including microaggressions [51], vicarious racial discrimination [29], and racial biases [28]. Decades of psychometric research have produced dozens of measures of racial discrimination, with the understanding that none of them are entirely adequate since experiences of racial discrimination can vary across populations and can depend on time and place.

However, consistently, no matter which racial discrimination measure is used, there are always some people of color who will deny experiencing any racial discrimination. For example, Alamillo [1] documented the denial of racism among a sample of Hispanic Americans, which suggests that ideologies or world views could lead some to overlook racism. Other possible explanations include perceiving racism vis-à-vis the experiences of other racial groups. For instance, O’Brien [31] argued that Latino and Asian Americans find themselves in the “racial middle” between a Black-White binary, whereby complex racial situations lead them to deny experiencing racism. Further, other explanations allude to difficulties discerning what constitutes racial discrimination. For instance, Rojas-Sosa [40] found that Latino students hesitated to recognize that they experienced racial discrimination partly because they questioned the motives behind the incidents and whether the experience qualified as racial discrimination. Roth [42] studied Dominican Americans and Puerto Ricans and suggested that they may have denied racial discrimination by only considering overt forms while tending to blame the person experiencing the discrimination. Along these lines, Lewis and colleagues [27] point out that minimization biases can result from perpetrators denying that discrimination occurred and can also result from the subtlety and ambiguity of discrimination.

The psychometric journey to arrive at items and instruments that can accurately capture a person’s experiences of racial discrimination has been long and challenging, and misreporting racial discrimination can result in a biased literature on racism and health. Specifically, individuals erroneously reporting no experiences of racial discrimination could dampened the estimated effects of racial discrimination on substance use and behavioral health in general. Based on the few qualitative studies that have addressed this topic, we sought to understand additional factors that may influence whether people of color deny experiences of personal racial discrimination, which by extension undermines potential associations between racial discrimination on substance use.

## Methods

### Procedures

We conducted this study under the approval of the University of Southern California Institutional Review Board. Participants were recruited via social media ads (Facebook, Instagram, Craigslist, and Reddit), ResearchMatch (an online research panel funded by the National Institutes of Health [NIH]), and in-person recruitment (e.g., at campuses and vape/cannabis retailers in Southern California). Participants completed an online screener, and study personnel validated their responses by contacting them via phone. All participants completed an electronic informed consent form via REDCap prior to the focus group. Focus groups were conducted online via Zoom between July and October 2023. Each focus group lasted approximately 60–90 min. Focus groups were stratified by gender and race/ethnicity, except for one general group containing a mix of participants in terms of race/ethnicity and gender. Participants received a $50 gift card upon completion of the focus group. Authors HO and MS are scholars trained in qualitative methods, and they conducted the focus groups with the assistance of support staff and students. The interviewers did not have any prior relationships with the participants and thus introduced themselves at the beginning of each focus group.

### Positionality

Mindful that our identities can shape our research, we would like to disclose information about our backgrounds. With respect to gender, seven authors self-identified as women and two authors as men. With respect to race, five authors self-identified as Asian American, two as Persian American, one as Black American, and one as White American.

### Participants

A total of 43 BIPOC adults participated in seven focus groups (ranging from five to nine participants per group). The eligibility criteria included: 21 to 44 years of age; identify as Black/African American, Hispanic/Latino, or Asian/Asian American; self-report current use of two or more of the following substances (alcohol, cigarettes, e-cigarettes, or cannabis); and reside in a U.S. state where recreational adult cannabis use is legal. A description of the participants for each focus group is available in the Supplemental Materials. In accordance with the Journal Article Reporting Standard for Race, Ethnicity, and Culture, we acknowledge that terms to describe race and ethnicity are used differently across contexts and that norms and practices for communities are evolving. We consulted with our community advisory board, and opted to use the term “people of color,” with the understanding that this term may not be universally accepted. All participants were permitted to select into the focus groups that they self-identified with in terms of race/ethnicity and gender. The recruitment strategy is described in Figure S1. We intentionally recruited from cannabis/vape stores to increase the likelihood of recruiting participants who couse. The focus groups varied in size (Table S1) and each focus group tended to have participants who were young adults and middle-aged adults: pilot focus group (*N* = 9; aged 22–40); Black male (*N* = 5; aged 31–42); Black female (*N* = 7, aged 22–41); Hispanic male (*N* = 6 aged 28–44); Hispanic female (*N* = 6 aged 27–43); Asian male (*N* = 5 aged 27–36); and Asian female (*N* = 5, aged 21–42).

### Focus Group Discussion Guide

The focus groups were conducted with a semi-structured focus group discussion guide. The guide included prompts such as “Please describe a time when you experienced discrimination in your life” and questions such as “Do you see any connection between racial discrimination and your substance use?” We showed respondents a slide listing the items of the Everyday Discrimination Scale [50] and invited them to reflect on the items. Examples of items from the scale included, “You are treated with less courtesy than other people are,” and “You receive poorer service than other people at restaurants or stores.” While focus groups were semi-structured, each focus group granted respondents the freedom to answer questions while also bringing up topics spontaneously. The respondents only participated in a focus group once over Zoom, and we did not present transcripts to respondents for member checking. We utilized the automated transcription function of Zoom to generate transcripts, which were then checked by professional transcribers.

### Analysis

Our focus groups collected 11 hours of audio recordings, yielding approximately 420 pages of transcripts. Focus group facilitators also completed “memos” after each focus group, yielding detailed descriptions that captured our immediate contextual understanding from each focus group. These memos and transcripts provided the raw data that we systematically analyzed for emergent themes. For the purposes of this paper, we examined themes relating to denying and minimizing racial discrimination. We drew from Braun and Clarke’s phases of reflexive thematic analysis [8], including familiarization with the data, generating codes, searching for themes, reviewing themes, and defining themes. We operated under constructionist and critical theoretical assumptions and viewed the data through an inductive lens. We wrote memos after each focus group and used our observations to link the focus group data to codes and themes. To arrive at themes, we drew codes from across focus groups, which were stratified by race/ethnicity and gender. Naturally, themes did not comprise an equal distribution of codes from across focus groups. However, the absence of codes from a particular focus group should not be taken to mean that the theme is irrelevant for that specific race/ethnicity or gender. Conversely, the presence of codes in a focus group should not be taken to mean greater salience of the theme for that race/ethnicity or gender, given that the emergence of a given code/theme is in large part a function of how questions are asked and how conversations evolve. That is, a code could very well have emerged in any focus group had the interviewers asked the questions in a different way (e.g., more explicitly). To frame our inductive findings, we consulted existing literature. We coded all transcripts by hand and discussed the emerging themes as a research team. We presented the themes to the community advisory board to check for resonance and credibility.

## Results

After conducting six focus groups, we noted that some respondents felt as though they did not experience racial discrimination, which created a disconnect between racial discrimination and substance use in the participants’ minds. In fact, some of the participants expressed frustration that the facilitators were probing about how respondents coped with discrimination. Then there were some respondents who reported racial discrimination but did not see that it was related to substance use in any way. Here, we present five emerging themes about what factors might contribute to these responses, which are (1) minority comparison effect; (2) the drowning out effect; (3) the diversity and racial composition of the context; (4) passing as White; and (5) covertness of racism.

### Theme 1. Minority Comparison Effect

One possible factor that may contribute to people denying racial discrimination may be the idea that people often compare their experiences of racial discrimination with the experiences of other ethno-racial minority groups that are more blatant and seemingly more disadvantageous. For example, some Asian male respondents minimized the seriousness of their own experiences of racial discrimination because they compared their experiences with those of Black Americans. One respondent stated:

I’m indifferent [immune] to it, right, because it does not negatively impact my employability. It does not, I mean, to me it doesn’t affect my life in any way because I’m not viewed negatively… So, it’s something that didn’t bother me…

[Asian Male, 27]

One Latino respondent recognized the discrimination of other ethnic groups and diminished his own experience by comparison:

Oh, my African American friends feel discriminated. Oh, my Asian friends feel discriminated and everything. The good thing about being Latino, I feel anyway, from my experience as well, too, that we’re in the middle of what White people, Black people experience. We’re in the middle. So, just because we’re in the middle, we’re not really involved in that situation. At least, once again, that’s my experience. My experience. And that’s why I love being Latino.

[Hispanic/Latino Male, 34]

We reasoned that if individuals did not recognize racial discrimination, then it was unlikely that they would see racial discrimination being related to substance use. An Asian male respondent seemed to illustrate this finding when discussing the socioeconomic status of Asian Americans:

No, I don’t think there is a connection between substance use and discrimination. The discrimination we get is because we are considered to be very hard working and successful. Chinese Americans, if you see the statistics, Chinese American families, their average median income is the highest and in the number two position it’s Indian American communities. Then I think it’s Filipino-American and then Korean Americans, then Vietnamese. You can check that later on, but I think it’s our success which brings discrimination to us. But I would say there is no relation between substance and [discrimination] --.

[Asian Male, 29]

In this response, we saw that the respondent did not regard positive stereotypes of Asian Americans to be a form of racial discrimination, which may have been linked to the failure to see any connection between racial discrimination and substance use.

### Theme 2. Drowning Out Effect

Another theme that emerged is that racial discrimination was not perceived to be especially impactful beyond other existing life stressors as they relate to substance use (e.g., such as financial stressors). One biracial gender-fluid person who chose to join the Black male focus group stated:

I think that when we say discrimination, what we’re really naming is stressors in general, and I think that a lot of people deal with a lot of different types of stress… I think that again, it just depends on the individual, right. I don’t know if it is based on race. It may be more of a socioeconomic thing, like your class, right, so it’s easier for you to go get a beer or smoke some weed than to go maybe get therapy, if you don’t have healthcare or if you don’t have access to healthcare

[Black Bi-racial American gender fluid person, 36]

In this response, we found an implicit acknowledgement that racial discrimination and substance use are related; however, the respondent cast the racial discrimination and substance use connection within a larger socioeconomic framework while de-emphasizing the racial nature of discrimination.

Other respondents acknowledged that life stressors in general were linked to substance use (e.g., coming home from a long day of work to open a can of beer), and some respondents felt that other aspects of their intersectional experiences were more likely the target of discrimination as opposed to race (e.g., being a parent of a child with disabilities, being a sexual minority).

### Theme 3. Diversity and Racial Composition of Context

Another contributing factor that may make it less likely for a person to report racial discrimination is the fact that people can live in ethno-racially diverse social contexts where one is less likely to encounter overt racial discrimination. One Latina woman stated that she “never” experiences racial discrimination because all her friends are diverse:

And I said, I’ve never felt in any way, shape or form discriminated or put down or anything. In fact, I have a lot of friends that are like me. They’re half Hispanic and they’re half White, right? So, where I live, it’s diverse. So, it’s very common.

[Hispanic/Latino Female, 27]

A Black Biracial man living in Buffalo, New York, described working for a diverse company where he currently does not experience much discrimination in contrast to his childhood.

I remember when I was a lot younger, I would experience it a lot more, just because the area that I went to school or grew up in, most everybody was White, I was really the only person of color, so growing was a little more difficult, people like just assuming things, you know. It’s hard to explain, it’s just like a feeling, but in my adult life, especially where I work is very diverse, so I don’t really experience it too much now, but definitely when I was a lot younger.

[Black Biracial American Male, 31]

In addition to the diversity of context, the racial/ethnic composition of the social environment also seemed to be tied to denying racial discrimination. One Latina woman described experiences of racism when she lived in a predominantly White city versus when she moved to Los Angeles:

But what I can relate to is like even growing up, you know, I lived a lot of years in Anchorage, Alaska where predominantly it’s a White community…But now living in Los Angeles where my surroundings are mainly Hispanic, I can go up the street and order my food in Spanish…

[Hispanic/Latina Female, 32]

Here we found that when she lived in a predominantly White area, she felt that she had experienced racial discrimination; however, she seemed to feel less ostracized in an area with higher ethnic density of Hispanic people.

Similarly, one Black female mentioned that in general, she does not experience discrimination in New Orleans, because the majority of the population is Black:

I will say, okay, so like in New Orleans. New Orleans is primarily Black, so you don’t really experience too much racism…But for the most part, you don’t really experience that much discrimination.

[Black American Female, 35]

However, the respondent also clarified that during certain parts of the year (e.g., during Mardi Gras), Black people can face discrimination when more White people come into town. She also mentioned that what was true for New Orleans (where there is high density of Black people) was not necessarily true for other parts of Louisiana.

### Theme 4. Passing as White

The focus group contained several multiracial individuals who felt that they did not experience very much racial discrimination because they presented as White, or because they had lighter skin tone, which may have granted them privilege. One Asian female stated she did not experience discrimination due to her appearance, but then also acknowledged that disclosing her ethno-racial identity could then bring about racial discrimination:

I would say that because I don’t present that much as a woman of color, I don’t experience discrimination, I guess at first, …but once I start to reveal parts of my identity, like being Filipino, I think that’s when the weird stuff starts happening for me.

[Asian Female, 21]

Another Latina woman described a moment when she could have attributed an event to racial discrimination, but reasoned that she did not look Mexican:

…I grew up in Washington State and there I was pulled over while not speeding or doing anything a couple of times, and my mom was always like, because you’re Mexican and I was like, ‘Mom, I don’t even look like Mexican.’”

[Hispanic/Latina Female, 38]

A Latino man mentioned his complexion, and described how he is treated more favorably because he did not have a darker skin tone:

…They’re like no, no, like they think it’s going to be a darker person you know? And to me I’m kind of like, no like we come in all shape and sizes, and colors. I think of when I answered like ads or when I answer emails like I put a picture of myself, not like to like be vain, but for people are like -- that’s racists it sounds like, “Oh like he’s not a like dark Mexican, like he’s not going to rob me.”

[Hispanic/Latino Male, 40]

Other examples included multiracial individuals intentionally passing as White by bringing a White parent to doctor appointments. In these accounts, the respondents suggested that while they did not often encounter racial discrimination, they acknowledged that racial discrimination does happen to other people in their communities.

### Theme 5. Covertness of Racism

Some respondents mentioned that racism was difficult to see plainly, and therefore it was difficult to be certain that one had experienced racial discrimination. One woman stated that people used to hold racist beliefs privately, though this has changed in recent years:

But in the other parts of Louisiana, oh boy, look out, and I will say I think that started changing after the 2016 election because down here in Louisiana people had their racism, but they kept it quiet and they kept it hush-hush, and then in 2016, they just stopped caring. They felt justified. They felt seen and heard. So they really started letting that come out, and I bet, it’s not just down here. I bet that’s in a lot of different places, but I’ll say like that’s when I started noticing that shift.

[Black American Female, 35]

Multiple respondents shared experiences of being singled out, receiving unwanted attention during class, or being teased but were uncertain whether they could be considered racial discrimination per se. Along these lines, one Black Biracial gender fluid person stated that they sensed racial discrimination mainly through passive-aggressive behaviors and “covert” discrimination:

…There’s a lot of microaggressions that happen in California, there’s a lot of passive-aggressive behavior in general, versus where I’m from, and so the ways in which you are able to cope, identify, and deal with discrimination here is different because it’s a lot of times much more covert, so yeah. …I think a lot of discrimination and a lot of microaggressions towards Black, Indigenous people, people of color, and just marginalized people in general, I think a lot of it is very coded now. It’s not like people are walking around calling each other ‘N word’ –

[Black American Male, 36]

Similarly, one Asian male also expressed this sentiment by stating that people often make racist remarks “behind closed doors,” and that people are “more careful about it,” meaning people are not as open or public about their racism [Asian Male, 27].

### Issues That Arose During the Focus Groups

In addition to the five themes, we also observed two main issues that emerged while running the focus groups, which were (a) individuals hesitated to state that they had experienced racial discrimination or contradicted themselves, and (b) individual’s responses were sometimes dependent on the orienting cues of the facilitator.

#### Hesitation and Contradictions

In conducting focus groups, we reflect on some of the inherent challenges and uncertainties that emerge from qualitative data collection, analysis, and interpretation. Some respondents expressed uncertainty about whether they had experienced racial discrimination. One Asian male respondent expressed this uncertainty after providing an example of being mistreated:

In this career fair and I was standing how anyone stands to talk to a person. And then the two people who were in front of me, they were, they were treated pretty nicely, but then I could see they were having a laugh and so and so. And then, my turn comes up. I introduce myself and she’s like not even interested in talking to me. She was like okay, give me your resume and she was in a lot of hurry. And behind me there were like six more people. I was like if she could give time to these two people in front of me, though, why not me? So, that was kind of very -- so, in terms of these nine things [items on the Everyday Discrimination Scale], I would say it was rude and she treated me like as if I didn’t matter. And I don’t know if that was my like my name or my appearance or something, which triggered her. I’m not sure.

[Asian Male, 29]

In other words, respondents sometimes could identify moments when they were treated unfairly but were uncertain about whether they could call it racial discrimination. However, later, the respondent mentioned a specific form of discrimination that is often tied to race, which is language discrimination:

I have also read that a lot of recruiters when they talk to you over the phone they judge you based on your accent, too. So, if you’re not a very smooth talker then they don’t like you. That’s what I’ve read…they judge you pretty early, and that’s what ruins everything.

[Asian Male, 29]

We note that respondents sometimes appeared to contradict themselves. Contradictions are normal for any thought process, especially when articulated for the first time in a social setting without any premeditation. For instance, one Asian American male who minimized the impact of racial discrimination also stated that he was steered away from a career due to racism:

…I actually left my PhD program. But during my PhD program, I mean, I come from a public school which I won’t name, and my advisor told me that – it’s a school which is different from what where you are from which is highly selective and very White. So, my professor, my advisor specifically told me I would not make it because the Ivy League would not hire a [derogatory slur toward Asian people]…

[Asian Male, 27]

He went on to describe how his field was predominantly White, and that he witnessed people making racist remarks throughout his education and career.

#### Orienting Cues

When asked outright about whether respondents experienced racial discrimination, some initially stated that they had not experienced any racial discrimination recently. However, when prompted with specific examples and contexts, respondents sometimes changed their answers. A Black Biracial man stated that he did not experience any discrimination, stating “In my adult life, especially where I work is very diverse, so I don’t really experience [discrimination] too much now,” But when he was specifically prompted about his Biracial identity, he then stated that he experienced racial discrimination “all of the time,” albeit not a severe form of racial discrimination:

Oh yes, yeah, so it’s almost every day, actually, because I guess I don’t have like one distinctive look, so people are always wondering what I am, but it’s usually like the first thing they ask me before they even ask like, “Hello, hi, what’s your name?” They’ll say like, “What are you,” and that is sort of offensive, especially if I don’t know the person, and to me I’m just like, “Why does it matter what I am? What does that have to do with the topic or why we’re here talking?” So that can be a little off-putting, but for the most part, I don’t experience any like aggressive discrimination or anything like that, or name-calling.

[Black Biracial American Male, 31]

## Discussion

### Main Findings

Across all three ethno-racial groups of adults who use substances, we found some respondents denied personally experiencing racial discrimination or hesitated to identify their experiences as racial discrimination and thus did not necessarily see racial discrimination related to their substance use. This is important to consider in light of numerous studies showing racial discrimination increases risk for substance use [2, 17, 21, 26, 41], some of which argue that stress pathways underlie the associations between racial discrimination and substance use. Our findings raise the question of whether these stress pathways are activated for people who do not recognize experiences of racial discrimination or are not bothered by these experiences. Inductively, we noted themes that may help explain these responses, including a minority comparison effect; a drowning out effect; diversity and racial composition of the context; passing as White; and covertness of racism. These themes are connected and interactive in the lives of people of color. We also note methodologically that there were hesitation and contradictions in accounts (i.e., respondents both denying *and* endorsing experiences/impacts of racial discrimination) and that responses depended on orienting cues (i.e., needing to prompt individuals with nuanced examples of racial discrimination). Consistent with the challenges of studying racial discrimination [27], a complex set of factors facilitates and hinders people from readily recalling and identifying experiences of racial discrimination. However, understanding the minimization or denial of racial discrimination among people of color can invigorate future research to better capture risk factors for substance use outcomes.

### Explanation of Findings

In terms of the minority comparison effect, some respondents in our focus group felt that their experiences of racial discrimination paled in comparison to high-profile overt events that Black people experienced. For instance, in 2020, the deaths of George Floyd, Eric Garner, Breonna Taylor, Philando Castile, and numerous other Black Americans at the hands of police served as an impetus for the Black Lives Matter protests in a long overdue recognition of anti-Black racism enacted through law enforcement [11]. If these events are examples of racial discrimination, then other less severe experiences of racial discrimination could appear unworthy of consideration. Our focus group participants appeared to recognize that racial discrimination should be understood in terms of severity, seeming to suggest that certain types of racial discrimination may be minimized or discounted. Asian American men perceived that the racial stereotypes against them were generally positive, and therefore had minimal consequences. The comparisons between Asian Americans and other racial groups are not a new phenomenon, yet studies have shown the insidious impacts of the Model Minority Myth and positive stereotypes can have on mental and behavioral health [23–25]. Interestingly, respondents did not bring up the surge of Anti-Asian hate crimes and xenophobia following the COVID-19 pandemic, documented in other studies [45, 52].

In terms of the drowning-out effect, some respondents did not feel that the racial discrimination was very salient or impactful on their substance use when juxtaposed next to the host of other stressors in their lives, specifically socioeconomic factors. This suggests that people may not see that their financial stressors or socioeconomic status are necessarily related to a larger system of racism, standing in contrast to academic conceptualizations of racism that span multiple levels, including opportunity structures [44], intergenerational transfers of wealth [9], discrimination in markets and financial institutions [36], neighborhood level factors [13], and so forth. Bourabain and Verhaeghe [7] found that studies of everyday racial discrimination tended to take on an individual perspective, while overlooking the micro–macro link of everyday racism.

In terms of diversity of context and racial composition of the context, some respondents denied or minimized racial discrimination because they felt that they inhabited tolerant social environments where they did not encounter such events. Examples included having a diverse group of friends, or living in a politically liberal or diverse city where overt acts of racial discrimination were publicly condemned, although some participants discussed historical/temporal contexts that changed the level of overtness. Certainly, residing in neighborhoods with high ethnic density may be protective for health in some contexts [4], as can having a diverse social support system [10, 43]. Findings suggest that propinquity may shape social experiences [39] and that social and geographic location may partly determine the likelihood of experiencing and reporting racial discrimination.

In terms of passing as White, there is a sizable body of literature on “passing,” which is defined as when a member of one racial group is accepted or perceived as a member of another racial group [20]. Passing has been associated with advantages in various contexts and may result in better health outcomes [15]. Some have suggested that having some ancestry tied to Europe (and White-appearing features) may bestow benefits by way of White privilege [48]. However, recent studies suggest multiracial individuals have some elevated risk for mental and behavioral health problems, though much depends on how a person identifies and is viewed/treated by society [32, 33]. It is possible that multiracial individuals who are racialized as White may not report experiences of racial discrimination. However, some respondents in our study acknowledged that while they personally did not experience racial discrimination, that other people in their ethno-racial group did.

Finally, in terms of covertness and subtlety of racism, scholars have long pointed to the covertness and invisible nature of racism [6]. While blatant racial discrimination like hate crimes tend to be easier to recall, there are numerous aspects of racism that operate outside of plain view, for example, through structures and institutions [27]. Some of these aspects may be revealed through audit studies [14], and other objective measures of racism, but these notions of racism may not come immediately to mind on a survey or in an interview. With that being said, racial discrimination can also be subtle, which has led to a wave of research on microaggression [35] and racial biases [46] that link to greater substance use. People may not be aware of the multiple invisible ways they are impacted by racism or may hesitate to label their experiences as racial discrimination [27]. We emphasize that even in theory if a person of color were to never perceive interpersonal racial discrimination, this does not mean that the person has been unaffected by racism in some way.

### Interactions of Themes

While we presented five discrete themes, the reality is that the themes are likely connected and interact a great deal in the day-to-day lives of the respondents. For instance, respondents across multiple focus groups suggested that certain diverse contexts may allow for covert forms of racism to persist—that in diverse spaces, it may be rare occasions that one will be overtly discriminated against, giving way to more subtle or invisible forms of discrimination that occurs ‘behind closed doors’. Another example of interaction of themes is that the “minority comparison effect” may be explained to some degree by racial inequities, which determine the extent to which people experience a drowning out effect. One Asian American man stated that Asian Americans had higher socioeconomic status, while a Black American gender fluid person suggested that financial strain can be more salient than the stress of racial discrimination. However, the likelihood of experiencing financial strain stems from systemic racism, which was not explicitly addressed by the participants, but could explain the racial patterning that invites minority comparisons. A final example of an interaction is that passing as White could be strongly correlated with minority comparison. We noticed from both the men and women’s Latino focus groups that respondents who self-reported appearing ‘White’ felt that they had not experienced as much discrimination as those who presented with darker features or with phenotypes that more strongly signaled Latino ethnicity. In this instance, the themes interact and show that even within ethno-racial groups that there can be comparisons (e.g., by skin tone) that lead to the discounting of racial discrimination.

### Limitations

First, our findings only scratch the surface of providing possible reasons why some people of color who use substances do not report experiencing racial discrimination or do not report changes in their substance use when they experience racial discrimination. More mixed methods research is warranted. Second, in terms of sampling, our findings are in part a function of our recruitment strategy (we oversampled in the Bay Area and Los Angeles, California), since the selection of each individual and the unique configuration of each focus group can influence the valence or “vibe” of each group. Future studies can strategically sample from across various social and geographic locations, and across intersections of identity. Third, since focus groups are subject to group dynamics, the responses of each participant are understood to be at least partly influenced by the presence of other members of the focus group and their responses. These dynamics (and the possibility of social desirability) are inherent and leveraged in the method. Fourth, as stated earlier, there were contradictory and inconsistent accounts, which is common when people are asked to answer questions extemporaneously. However, these contradictions and inconsistencies may be areas for future research. Finally, we did not ask about specific aspects of ethno-racial identity (salience, centrality, regard) or ask deeply about acculturation (other than requiring all respondents be English speaking), and this may have shaped whether people were more or less likely to report racial discrimination.

### Implications

Findings from our study may have implications for practice and research. In terms of practice, both clinical and community, we maintain that our coordinated interdisciplinary efforts should aim to dismantle racism in its many forms, which would improve population level behavioral health among racially and ethnically minoritized communities. These efforts should dovetail with broader public health strategies to prevent and treat substance use in the general population, as minoritized communities would likely benefit from these strategies if given proper access and tailoring if needed.

In terms of research, our findings suggest researchers should invest in carrying out more activities that align definitions and notions of racism with public/community understandings of racism. We acknowledge the possibility that there may be “top-down” conceptualization of racism—that most people do not have an intimate knowledge of the various models and frameworks that researchers employ in scholarly or academic circles. On one hand, it is important for academics to educate the public on the many insidious ways in which racism impacts the lives of people of color. However, at the same time, it is critical that academics dialogue with and learn from communities about racism to ensure concordance around definitions, concepts, and measures [34]. Dedicating time during data collection (surveys and interviews) to discuss how race and racism are defined may be useful.

Another research implication is that measures need to be updated to keep up with the evolving forms of racial discrimination. Developing measures for racial discrimination requires tremendous effort, especially validating the measures for various populations. Researchers increasingly wish to modify the wording of existing items, add new items, and change the response options, but are unable to, given that this is time consuming and costly. For example, many studies use the Everyday Discrimination Scale, which contains the item “people assume you are not smart.” Our focus groups show that some Asian Americans may deny this item but would endorse the converse, which would look something like “people assume you are good at math and science” or other positive stereotypes. However, modifying discrimination measures, even slightly, runs the risk of creating potential for measurement biases. An alternative strategy is to administer multiple measures to capture more aspects of racial discrimination (e.g., online racism, microaggressions, perceptions of group-level discrimination, vicarious discrimination, inconsistent racial biases for multi-ethnic individuals). But the sheer number of measures can be burdensome to participants and impractical to administer. Still, our findings suggest that it may be important for surveys and focus groups to cue people to think about various instances where one might experience racial discrimination, even though the list of possibilities will never be exhaustive. Providing more specific examples, that are timely and context-specific, may be critical.

A final research implication is that subjective, individual-level measures of racial discrimination are just one way to understand the impact of racism, and findings should be compared with findings from studies that use objective, macro-level measures, such as neighborhood-level exposures, consumer behavior data [12], and auditing experiments [14, 38]. Our findings suggest that some people do not connect their experiences of racial discrimination to the larger institutional, cultural, structural, or systemic nature of racism. One possibility is that the likelihood of seeing these connections may depend on one’s awareness of racism as it manifests as social, economic, and political forces. It may be worthwhile to show respondents empirical evidence of how racism operates through socioeconomic status, policies, and institutions; however, this would likely produce response biases.

## Conclusion

A complex set of factors facilitates and hinders people from readily recalling and identifying experiences of racial discrimination, including the minority comparison effect, the drowning out effect, the diversity and racial composition of one’s environment, passing as White, and the subtlety of racism. Contradictions in accounts are not uncommon when recalling experiences of racial discrimination (or lack thereof) and orienting cues may be necessary. Understanding the denial of racial discrimination among people of color can guide future efforts to better capture these experiences in relation to polysubstance use.

## Supplementary Material

Supplemental Materials
