# Supplementary material for: Denial of Personal Racial Discrimination and Its Impact Among People of Color Who Use Substances: Implications for Measuring Racial Discrimination in Substance Use Research
Source: J Racial Ethn Health Disparities. 2024 Jun 10;12(4):2091–102. doi: 10.1007/s40615-024-02033-w (PMC11687556; doi:10.1007/s40615-024-02033-w)
Supplement: Supplementary file 1 — Supplementary file1 (PDF 891 kb) [file 40615_2024_2033_MOESM1_ESM.pdf]

**Supplemental Materials**
**Figure S1.**

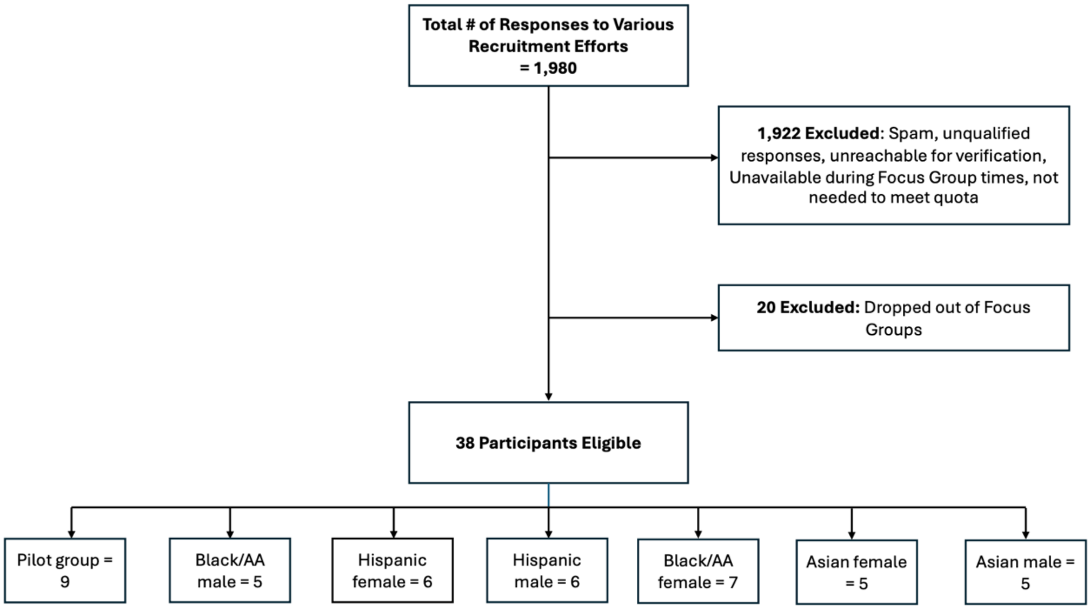

**Table S1. Demographic characteristics of participants by focus group.**

| Focus Group | Race | Gender | Age |
|---|---|---|---|
| **Pilot** | | | |
| | Hispanic | Male | 30 |
| | Black/African American, White | Female | 24 |
| | Asian | Male | 24 |
| | Hispanic | Male | 40 |
| | Black/African American | Female | 40 |
| | Asian, White | Male | 22 |
| | Black/African American | Male | 29 |
| | Asian | Male | 23 |
| | Hispanic | Male | 34 |
| **Black/African American Male** | | | |
| | Black/African American | Male | 36 |
| | Black/African American | Male | 32 |
| | Black/African American | Male | 36 |
| | Black/African American | Male | 42 |
| | Black/African American | Male | 31 |
| **Black/African American Female** | | | |
| | Black/African American | Female | 41 |
| | Black/African American | Female | 32 |
| | Black/African American | Female | 36 |
| | Black/African American | Female | 32 |
| | Black/African American | Female | 35 |
| | Black/African American | Female | 43 |
| | Black/African American, Asian | Female | 22 |
| **Hispanic Male** | | | |
| | Hispanic | Male | 37 |
| | Hispanic | Male | 44 |
| | Hispanic | Male | 28 |
| | Hispanic | Male | 40 |
| | Hispanic | Male | 34 |
| | Hispanic | Male | 37 |
| **Hispanic Female** | | | |
| | Hispanic | Female | 38 |
| | Hispanic | Female | 31 |
| | Hispanic | Female | 27 |
| | Hispanic | Female | 37 |
| | Hispanic | Female | 32 |
| | Hispanic | Female | 43 |
| **Asian Male** | | | |
| | Asian | Male | 36 |
| | Asian | Male | 29 |
| | Asian | Male | 25 |
| | Asian | Male | 27 |
| | Asian | Male | 22 |
| **Asian Female** | | | |
| | Asian | Female | 21 |
| | Asian, Native Hawaiian/Pacific Islander | Female | 28 |
| | Asian | Female | 42 |

| | | | |
|---|---|---|---|
| | Asian | Female | 24 |
| | Asian | Female | 39 |

| | CSUF Campus | Email | Facebook | Instagram | Reddit | Craigslist | Google Ads |
|---|---|---|---|---|---|---|---|
| Recruitment Type | In Person | Email | Social Media paid ad and posting on SSRC page | Social Media paid ad and posting on SSRC page | Social Media paid ad and posting on research subreddit forums | Paid classified ad | Search engine paid ad |
| Reach | CSUF campus population | CSUF Chicano studies students[1]; SSRC employees[2]; | USA | USA | USA | USA | USA |
| Paid Ad Campaign Search Criteria | - | - | 21 – 44 years old; | 21 – 44 years old; | 21 – 44 years old; keyword search for alcohol, tobacco, vapes, smokes shops, and dispensaries; | Searching for employment ads (in line with Craigslist ad regulations) | 21 – 44 years old; keyword search for alcohol, tobacco, vapes, smokes shops, and dispensaries |
| Paid Campaign Dates | - | - | 08/28/23 – 09/04/23 | 07/10/23 – 08/28/23 | 07/26/23 – 08/15/23 | 08/25/23 – 10/15/23 | 07/24/23 – 08/28/23 |
| Unpaid Campaign Search Criteria | Recruited to take survey by SSRC employees on Titan Walk or viewed flyers posted on campus | Students enrolled in Chicano studies; current employees of the SSRC | Follow the SSRC page | Follow the SSRC page | Follow CSUF, Los Angeles, Orange County, research participation subreddits | - | - |
| Unpaid Campaign Dates | Titan Walk: 09/12/23<br>Flyer: 09/04/23 – 10/02/23 | CSUF: 08/24/23<br>SSRC: 08/14/23 | 09/12/23 | 07/10/23 | 08/28/23 | - | - |

[1] Email sent by Chicano studies professors.
[2] Email sent by SSRC using general ssrc@fullerton.edu account.

NOTES

a. A total of 1,980 responses were recorded from all the different recruitment methods.
    I. 1,922 responses were not selected due to being spam responses (i.e. scammers), having unqualified survey responses, being unreachable for verification of responses, being unavailable to attend the focus group on the designated date and/or time, or not being needed to meet the quota for the focus group.
    II. 20 individuals dropped out of the focus groups.
    III. 38 individuals participated in the focus groups, with nine in the pilot and 29 in the actual discussions.
b. Participants were placed into the groups that were associated with the race/ethnicity and gender reported in the survey responses. In instances where someone reported being multi-racial with White being one of the categories selected, they were placed into the other racial category they selected. In instances in which someone selected two or more qualifying race/ethnicities, they were placed in the FGD where their participation was most needed to meet the quota. Individuals who reported a gender identity other than man, woman, transgender man, or transgender woman (i.e. non-binary, genderqueer) were not eligible to participate in the groups.
